# ​​Exploring the Efficacy of an Acute Coronary Syndrome Simulation Scenario for Fourth-Year Medical Students​

**DOI:** 10.7759/cureus.88968

**Published:** 2025-07-29

**Authors:** Heena Mansuri, Juliana Cazzaniga, Jenny Fortun, Gagani Athauda, Rebecca L Toonkel

**Affiliations:** 1 Medical School, Florida International University, Herbert Wertheim College of Medicine, Miami, USA; 2 Department of Cellular Biology and Pharmacology, Florida International University, Herbert Wertheim College of Medicine, Miami, USA; 3 Department of Internal Medicine, Florida International University, Herbert Wertheim College of Medicine, Miami, USA

**Keywords:** acute coronary syndrome, clinical skills, medical education, simulation-based education, transition to residency

## Abstract

Introduction: Acute coronary syndrome (ACS) is ​a critical medical condition characterized by a sudden reduction in blood flow to cardiac muscle. This condition encompasses unstable angina, non-ST-elevation myocardial infarction (NSTEMI), and ST-elevation myocardial infarction (STEMI), requiring rapid diagnosis and intervention for optimal outcomes. Despite advances in treatment, ACS remains a leading cause of morbidity and mortality worldwide.​ Thus, it is essential to ensure that graduating medical students possess the requisite knowledge and confidence to effectively identify and manage patients with ACS as they transition to residency.​ ​Methods: To ensure the readiness of graduating medical students to recognize and initiate management of ACS, we developed and implemented a 45-minute simulation-based scenario during the CO 2023 transition to residency course at the Florida International University Herbert Wertheim College of Medicine (FIU-HWCOM).​ Knowledge gains were ​assessed​ through performance on voluntary pre- and post-session identical assessments consisting of seven multiple-choice questions. Pre- and post-session confidence and post-session satisfaction were assessed using voluntary 5-point Likert-style questions. Results: All graduating FIU-HWCOM medical students (n=120) participated in the simulation. Students who completed both the pre- and post-session assessments (n=50, 41.7%) were included in the study. Mean knowledge performance improved from 3.74 (SD: 1.14) to 5.34 (SD: 1.19) (p=0.0001) post-session. Confidence in recognizing acute cardiac ischemia increased from 3.88 (SD: 0.66) to 4.42 (SD: 0.67), while confidence in initiating management of acute cardiac ischemia increased from 3.54 (SD: 0.76) to 4.32 (SD: 0.65) post-session (p=0.0001). Overall satisfaction was high, with 94% of participants finding the session a valuable use of their time and 90% agreeing that participation will positively affect the care of patients presenting with chest pain. ​

​​Conclusion: ​Our findings suggest improvements in graduating medical students’ knowledge and confidence in recognizing and managing ACS. The session was well received by students and is easily adaptable for integration into curricula at other medical schools.

## Introduction

Acute coronary syndrome (ACS) is a critical medical condition characterized by a sudden reduction in blood flow to cardiac muscle, typically due to atherosclerotic plaque rupture or thrombus formation in the coronary arteries [1]. This condition encompasses a spectrum of cardiovascular emergencies, including unstable angina, non-ST-segment elevation myocardial infarction (NSTEMI), and ST-segment elevation myocardial infarction (STEMI) [1]. ACS is associated with high prevalence, significant morbidity and mortality, and substantial healthcare expenditures [1,2]. Swift and accurate diagnosis, followed by timely intervention, is essential for improving patient outcomes [2].

Given the prevalence and emergent nature of ACS [3], all physicians, regardless of specialty, must be prepared to recognize and initiate appropriate management for patients presenting with symptoms suggestive of ACS [4-6]. Early and accurate identification of ACS is critical, as timely intervention may reduce morbidity and mortality and can significantly impact patient outcomes [7]. This ability is particularly crucial for graduating medical students transitioning to residency, who may soon be the first responders to patients with chest pain in emergency departments, inpatient units, or outpatient settings. Furthermore, ACS management is heavily emphasized in medical licensing examinations, reflecting its clinical significance [8]. Yet, new residents often feel underprepared for managing acute cardiac emergencies, highlighting the need for targeted and effective active learning educational interventions during the transition from medical school to residency [9,10]. 

Medical education has evolved toward increased inclusion of active learning due to its proven benefits in improving student learning outcomes [11]. Various active learning modalities exist, including case-based learning, flipped classroom, and simulation-based education [12-14]. Among these, simulation-based learning has become increasingly prominent in medical education, particularly for teaching emergency and critical care scenarios [13]. Simulation offers several unique advantages: it provides an immersive, experiential learning environment; allows students to practice high-stakes clinical scenarios without risk to patients; and enables immediate feedback on clinical reasoning and decision-making [15,16]. For emergent conditions like ACS, simulation-based education has been shown to improve both knowledge retention and clinical performance while developing essential team communication skills that are crucial in emergency settings [17, 18].

A search of the medical education literature reveals that ACS-related simulation implementation for graduating medical students is limited. Reported simulations vary in their structure, implementation timing, and evaluation methods [19-23]. To our knowledge, none of the reported studies have evaluated the effectiveness of ACS simulation during the critical transition to residency period as graduating medical students prepare to assume the role of first responder.

To fill this gap, we developed and implemented a small group simulation-based learning activity for graduating fourth-year medical students designed to consolidate prior learning on the identification and management of patients presenting with ACS. This study aimed to evaluate the impact of a simulation-based ACS scenario on graduating medical students’ knowledge and confidence in recognition and initial management of ACS during the transition to residency.

## Materials and methods

Session design and implementation 

Participants in this study were graduating fourth-year medical students (Class of 2023) at the Florida International University Herbert Wertheim College of Medicine (FIU-HWCOM) enrolled in the required clinical skills transition to residency capstone course. The mandatory 45-minute ACS simulation was conducted in small groups of 5-7 students. 

The simulation began with students and a faculty facilitator seated around a conference room table while the standardized patient was behind a curtain. Faculty facilitators included physician faculty members from the Department of Graduate Education. Each facilitator has a robust clinical background, with an average of 10 years in clinical practice (range: 4-16 years). All facilitators had prior experience in simulation-based education and regularly participated in both undergraduate and postgraduate medical training. Students were provided with a “student information” sheet containing relevant patient information, while faculty facilitators were provided with a guide to ensure consistency in guiding the simulation encounter. The scenario involved a woman with a history of hypertension and diabetes mellitus who is hospitalized for treatment of lower extremity cellulitis and who goes on to develop chest pain. The student (playing the role of the intern on call) is called by the facilitator (playing the role of the nurse) to respond to evaluate the patient. ​In the first portion of the scenario, the patient was found to have ST depressions on the ECG, which the student must manage. In the second part of the scenario, a second student (playing the role of the covering intern) is called to respond to a recurrence of chest pain, now with the ECG showing ST elevations.​ Upon completion of the scenario, the facilitator then leads a debrief session using the Gather, Analyze, Summarize (GAS) model. ​The GAS model allowed students to first share their thoughts about the simulation, then analyze their actions and decisions, and finally, to summarize the key learning points and review learning objectives.​ During the debrief, facilitators ensure that all critical aspects of the scenario are discussed, and students are encouraged to reflect on any missed opportunities or alternate management strategies. 

Outcome measures and evaluation 

Baseline knowledge and confidence in recognizing and managing ACS were assessed through a voluntary pre-session assessment. The effectiveness of the simulation was then assessed with a voluntary post-session assessment (identical to the pre-session assessment). 

Pre- and post-session assessments were identical and accessed via QR codes linked to Qualtrics surveys (Qualtrics LLC, Provo, UT; Seattle, WA), and students were assured that participation was not required. The assessment consisted of seven knowledge-based multiple-choice questions (MCQs) on ACS and two Likert-scale questions (1-5, from strongly disagree to strongly agree) measuring confidence in recognizing and managing ACS. The post-session assessment included an additional two Likert-scale questions (1-5, from strongly disagree to strongly agree) assessing students' overall satisfaction with the simulation. 

Data analysis was conducted using STATA software (StataCorp LLC, College Station, TX). A paired t-test was performed to analyze the difference in knowledge scores between the pre- and post-session assessments. For Likert-style questions, a non-parametric Wilcoxon signed rank test was used to analyze changes in confidence and satisfaction. 

This study received institutional review board (IRB) exempt approval (IRB#112680) at Florida International University. 

## Results

**Figure 1:**
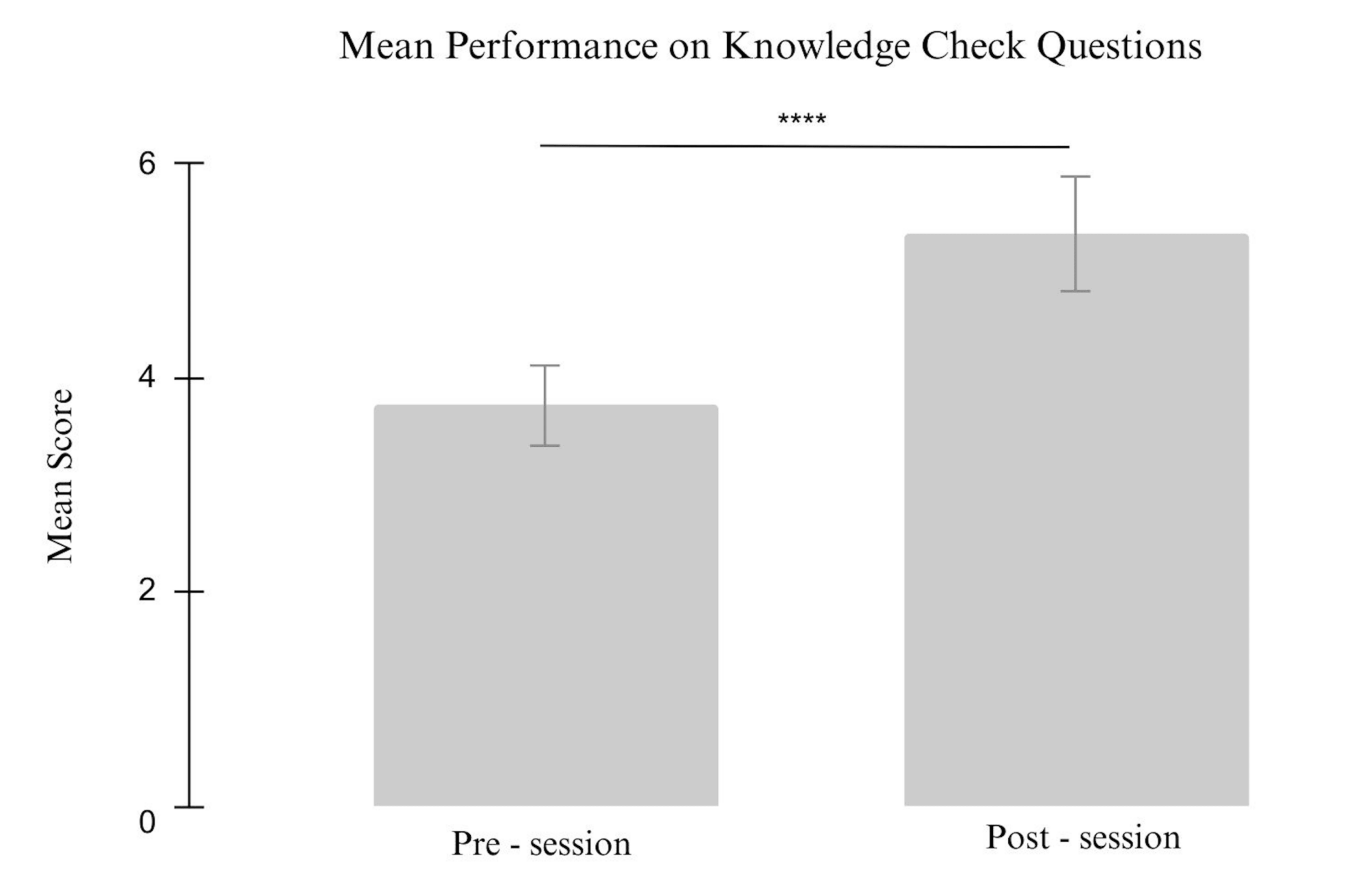
Comparison of mean performance on pre- and post-session knowledge-based questions​ p<0.0001, t(49) = 7.55, n=50 p-value calculated using paired t-test

Of 120 students participating in the simulation, 50 (42%) completed both the pre- and post-session assessments and were included in this study. A total of 120 students, the entire graduating class, were invited to participate in the intervention. Of these, 50 students voluntarily chose to attend and completed all required assessments. These 50 participants were ultimately included in the final analysis. As shown in Figure 1, the mean knowledge-based score was higher on the post-session assessment, compared to the pre-session assessment (pre-session, 3.74 (SD: 1.14) vs. post-session, 5.34 (SD: 1.19); p < 0.0001). 

As shown in Table 1, mean agreement with the statement, “I feel confident in my ability to recognize the signs and symptoms of acute cardiac ischemia" increased from 3.88 (SD 0.66) pre-session to vs. 4.42 (SD 0.67) post-session, (p < 0.0001) and mean agreement with the statement, “I feel confident in my ability to initiate management in a patient presenting with acute cardiac ischemia” increased from 3.54 (SD 0.76) pre-session to 4.32 (SD 0.65) post-session assessment (p<0.001). 

**Table 1:** Comparison of student confidence in recognizing signs and symptoms (p<0.0001) and initiating management (p<0.001) in patients presenting with ACS. p-values calculated using Wilcoxon signed-rank test

​Statement	​Pre-session ​assessment mean (SD)	​Post-session ​assessment mean (SD) ​	​p-value	z-score
​ ​“I feel confident in my ability to recognize the signs and symptoms of acute cardiac ischemia.”	​ ​3.88 (0.66)	​ ​4.42 (0.67) ​	​p<0.0001	z=4.8
​“I feel confident in my ability to initiate management in a patient presenting with acute cardiac ischemia.” ​	​3.54 (0.76)	​4.32 (0.65)	​p<0.001	z=5.23

Of the 50 students who completed the pre- and post-session assessments, 90% (n=45) students agreed or strongly agreed that participation would positively affect their care of patients presenting with chest pain. Additionally, 94% of students (n=47) agreed or strongly agreed that the session was a valuable use of their time (94%). 

## Discussion

​​ACS is an emergent and widely prevalent medical condition associated with significant morbidity and mortality.​ As a result, all physicians, including new residents, must be well equipped to recognize and initiate management for ACS. To ensure that graduating medical students have the requisite skills to do so, we developed and implemented a simulation-based ACS scenario for use during a transition to residency course. 

Our findings indicate that graduating medical students showed significant gains in knowledge in recognizing and initiating management for patients presenting with ACS. This is in agreement with previous studies that reported similar gains in knowledge and confidence following ACS simulations [19-23]. However, unlike previous studies that implemented ACS simulations during clerkships [20, 22, 23], our intervention specifically targeted the transition to residency period, which may explain the higher baseline knowledge scores in our cohort. Consistent with prior studies, student satisfaction with our session was also high [19, 20, 23]. While student satisfaction does not reﬂect learning, it remains an important outcome with regards to student engagement and participation, which are crucial to the success of active learning pedagogies [24]. 

Several factors may have contributed to the gains in knowledge and confidence observed. Our simulation-based approach aligns with Kolb's experiential learning theory, which emphasizes learning through concrete experience, reflective observation, abstract conceptualization, and active experimentation [25]. ​The hands-on nature of simulation allows students to engage with a realistic clinical scenario, reflect on their decisions during debriefing, conceptualize the principles of ACS management, and apply this knowledge in a controlled environment before facing similar situations in clinical practice.​ This experiential approach is particularly valuable for preparing students to navigate emergent conditions like ACS, where clinical reasoning under pressure is essential but difficult to teach through didactic methods alone. Additionally, the design of our simulation is unique, involving the management of both NSTEMI and STEMI, rather than focusing on a single presentation of ACS as with previous studies [19,21,23]. ​ ​ 

One limitation of our study is the relatively low response rate, 41.7% (50 of 120 students), and the subsequent possibility that the voluntary nature of the assessments may have led to response bias. It is possible that students who completed both the pre- and post-session assessments were more engaged with the material or had a higher baseline interest in cardiology or emergency medicine. These students might have performed differently from non-respondents, potentially inflating our measured effectiveness. However, the baseline knowledge scores (mean 3.74 out of 7) suggest that respondents were not necessarily starting with mastery of the subject. Future iterations could implement mandatory assessments to minimize this potential bias and capture a more representative sample of the student population. Limitations of the study include the lack of validation for the multiple-choice question (MCQ) assessment tool, as well as the absence of long-term follow-up or data on participants' clinical performance. In addition, while short-term knowledge retention was demonstrated, our study did not assess long-term knowledge retention or subsequent changes in clinical practice. While this could theoretically be accomplished through surveys of residency program directors or self-reporting by graduates, we acknowledge the significant practical challenges of such follow-up assessments. 

## Conclusions

Because of its high prevalence and its association with significant morbidity and mortality, it is essential that graduating medical students possess the requisite knowledge and confidence to identify and manage patients presenting with ACS. We designed and implemented a unique ACS simulation-based learning scenario for use during the transition to residency, which resulted in significant knowledge and confidence gains. Participation in an activity such as the one we describe may be adapted by medical educators at other institutions aiming to utilize active learning methods to teach the principles of diagnosis and management of ACS to fourth-year medical students as they near the start of residency.

## Figures and Tables

**Figure 2:**
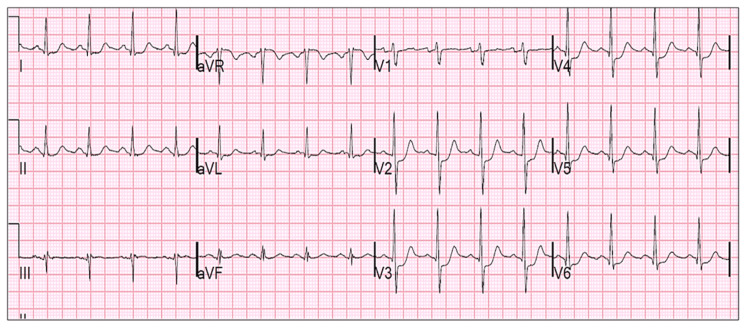
Acute anterior wall ischemia

**Figure 3:**
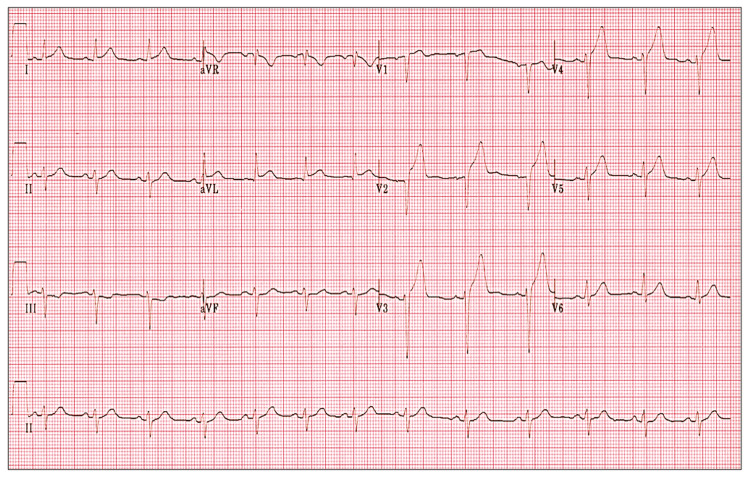
Acute anterior wall infarction

## Appendices

Pre-Session / Post-Session Questions

1. A 62-year-old man presenting to the emergency department with chest pain is found to have ST elevations on the EKG. Which of the following is the next best step in management? Alert cardiology to evaluate for urgent reperfusion (TPA or cardiac catheterization).

(A) Initiate IV fluids (NS or D5W) to prevent volume depletion.

(B) Administer warfarin to prevent further coronary artery thrombosis.

(C) Start oxygen via a non-rebreather mask to match cardiac oxygen supply and demand. 

Answer: Alert cardiology to evaluate for urgent reperfusion (TPA or cardiac catheterization).

2. Which of the following diagnostic studies should be ordered initially for hospitalized patients complaining of chest pain? (check all that apply)

(A) Vital signs, including O2 saturation

(B) Complete blood count

(C) Basic metabolic panel

(D) Troponin level

(E) EKG

(F) Echocardiogram

Answer: Vital signs including O2 saturation, Troponin, EKG

3. The EKG pictured depicts which of the following? (Figure 2)

(A) Acute anterior wall ischemia

(B) Old anterior wall infarction

(C) Acute inferior wall ischemia

(D) Old inferior wall infarction

Answer: Acute anterior wall ischemia 

4. Assuming normal or elevated blood pressure, which of the following medication combinations should be administered to a patient presenting with chest pain and ST depressions on the EKG?

(A) ASA, digoxin, heparin, platelet inhibitor

(B) ASA, digoxin, losartan, warfarin

(C) ASA, nitroglycerin, losartan, warfarin

(D) ASA, nitroglycerin, heparin, platelet inhibitor 

Answer: ASA, nitroglycerin, heparin, platelet inhibitor

5. The EKG pictured depicts which of the following? (Figure 3)

(A) Acute anterior wall ischemia

(B) Acute anterior wall infarction

(C) Old anterior wall ischemia

(D) Old anterior wall infarction

Answer: Acute anterior wall infarction

6. Which of the following correctly identifies the goal for time between ER arrival and the initiation of reperfusion treatment (TPA or catheterization) for a patient presenting with STEMI? 

(A) Less than 60 minutes

(B) Less than 120 minutes

(C) Less than 4 hours 

(D) Less than 24 hours

Answer: Less than 120 minutes

7. Assuming no contraindications, which of the following medications should be included at the time of  discharge for a patient who has undergone successful reperfusion after an STEMI? (check all that apply)

(A) ASA

(B) ACE inhibitor

(C) Platelet inhibitor

(D) Warfarin

(E) Statin

(F) Calcium channel blocker

(G) Beta blocker

Answer: ASA, ACE inhibitor, Platelet inhibitor, Statin, Beta Blocker

Satisfaction/confidence survey (Likert-style) 

Pre-survey

A. I feel confident in my ability to recognize the signs and symptoms of acute cardiac ischemia.

1. Strongly disagree

2. Disagree

3. Neutral

4. Agree

5. Strongly agree

B. I feel confident in my ability to initiate management in a patient presenting with acute cardiac ischemia.

1. Strongly disagree

2. Disagree

3. Neutral

4. Agree

5. Strongly agree

Post-Survey

A. I feel confident in my ability to recognize the signs and symptoms of acute cardiac ischemia.

1. Strongly disagree

2. Disagree

3. Neutral

4. Agree

5. Strongly agree

B. I feel confident in my ability to initiate management in a patient presenting with acute cardiac ischemia.

1. Strongly disagree

2. Disagree

3. Neutral

4. Agree

5. Strongly agree

C. Participation in the simulation session will positively affect my care of patients presenting with chest pain.

1. Strongly disagree

2. Disagree

3. Neutral

4. Agree

5. Strongly agree

D. The simulation session was a valuable use of my time.

1. Strongly disagree

2. Disagree

3. Neutral

4. Agree

5. Strongly agree
